# Forced arm use is superior to voluntary training for motor recovery and brain plasticity after cortical ischemia in rats

**DOI:** 10.1186/2040-7378-6-3

**Published:** 2014-02-14

**Authors:** Armin Schneider, Andreas Rogalewski, Oliver Wafzig, Friederike Kirsch, Norbert Gretz, Carola Krüger, Kai Diederich, Claudia Pitzer, Rico Laage, Christian Plaas, Gerhard Vogt, Jens Minnerup, Wolf-Rüdiger Schäbitz

**Affiliations:** 1 SYGNIS Bioscience, Heidelberg, Germany; 2 Neurology Department Bethel EVKB Bielefeld and Dept. of Neurology, University of Muenster, Muenster, Germany; 3 Ctr. for Med. Res., Fac. for Clin. Medicine, University of Heidelberg, Mannheim, Germany

## Abstract

**Background and purpose:**

Both the immobilization of the unaffected arm combined with physical therapy (forced arm use, FAU) and voluntary exercise (VE) as model for enriched environment are promising approaches to enhance recovery after stroke. The genomic mechanisms involved in long-term plasticity changes after different means of rehabilitative training post-stroke are largely unexplored. The present investigation explored the effects of these physical therapies on behavioral recovery and molecular markers of regeneration after experimental ischemia.

**Methods:**

42 Wistar rats were randomly treated with either forced arm use (FAU, 1-sleeve plaster cast onto unaffected limb at 8/10 days), voluntary exercise (VE, connection of a freely accessible running wheel to cage), or controls with no access to a running wheel for 10 days starting at 48 hours after photothrombotic stroke of the sensorimotor cortex. Functional outcome was measured using sensorimotor test before ischemia, after ischemia, after the training period of 10 days, at 3 and 4 weeks after ischemia. Global gene expression changes were assessed from the ipsi- and contralateral cortex and the hippocampus.

**Results:**

FAU-treated animals demonstrated significantly improved functional recovery compared to the VE-treated group. Both were superior to cage control. A large number of genes are altered by both training paradigms in the ipsi- and contralateral cortex and the hippocampus. Overall, the extent of changes observed correlated well with the functional recovery obtained. One category of genes overrepresented in the gene set is linked to neuronal plasticity processes, containing marker genes such as the NMDA 2a receptor, PKC ζ, NTRK2, or MAP 1b.

**Conclusions:**

We show that physical training after photothrombotic stroke significantly and permanently improves functional recovery after stroke, and that forced arm training is clearly superior to voluntary running training. The behavioral outcomes seen correlate with patterns and extent of gene expression changes in all brain areas examined. We propose that physical training induces a fundamental change in plasticity-relevant gene expression in several brain regions that enables recovery processes. These results contribute to the debate on optimal rehabilitation strategies, and provide a valuable source of molecular entry points for future pharmacological enhancement of recovery.

## Introduction

Stroke is the leading reason for permanent disability in the western world [1] and therefore one of the biggest and growing burdens for welfare systems. Improving care and treatment of stroke includes both development of new pharmacological strategies in the acute phase, as well as improvements in approaches that stimulate functional recovery after stroke by enhancing brain-inherent plasticity mechanisms. Up to now, despite several attempts at developing pharmacological means for enhancing recovery [2], the only measures to support motor recovery of stroke patients is physical therapy. Numerous questions arise around problems of optimal timing, frequency, and type of therapy used. Immobilization of the unaffected arm combined with physical therapy, the so called forced use paradigm, was shown to improve motor function of the impaired arm weeks after unilateral stroke in humans [3-6]. Not completely understood are timing and intensity of the training. This is important because experimental and clinical data indicate that an early overuse of the impaired limb could worsen functional outcome and exaggerate lesion size [7-10]. On the other hand, voluntary treatment paradigms where timing and intensity are controlled by the affected individual can also improve recovery and induce plasticity processes after focal cerebral ischemia.

Although it is clear that physical therapy enhances endogenous plasticity mechanisms of the brain the genomic mechanisms involved in plasticity changes after different means of rehabilitative training post-stroke are largely unexplored. Areas of the post-stroke brain that are of particular interest in view of plastic changes include the infarct-adjacent cortex (e.g. [11]), the contralateral homotopic cortex (e.g. [12]), and the hippocampus (e.g. [13]).

We have therefore set out to compare forced vs. voluntary measures of “rehabilitation” training in rodents regarding their outcome, and associated changes in gene expression, as clarification of these changes could allow a better understanding of the underlining mechanisms as well as a pharmacological targeting of mechanisms involved in rehabilitation-induced plasticity.

## Methods

### Ischemia model

All animal experiments were conducted in accordance with an institutionally approved protocol following the governmental authorities Landesamt für Natur, Umwelt und Verbraucherschutz Nordrhein- Westfalen. Male Wistar rats (Charles River; 280 to 320 g) were anesthetized with an i.p. injection of xylazine hydrochloride (Bayer, Leverkusen Germany) and ketamine hydrochloride (WDT, Garbsen, Germany). A PE-50 polyethylene tube was inserted into the right femoral artery for continuous monitoring of mean arterial blood pressure and blood gases. The right femoral vein was cannulated by a PE-50 tube for treatment infusion. During the experiment rectal temperature was monitored and maintained at 37°C by a thermostatically controlled heating pad (Föhr Medical Intruments, Germany).

Photothrombotic ischemia was induced in the rat parietal cortex [14,15]. Animals were placed in a stereotaxic frame, and the scalp was incised for exposure of the skull surface. For illumination, a laser was placed stereotaxically onto the skull 0.5 mm ventral to the bregma and 4 mm lateral from the midline. The skull was illuminated with a laser spot of 8 mm in diameter (G Laser Technologies) for 20 minutes. During the first 2 minutes of illumination, the dye rose bengal (0.133 mL/kg body weight, 10 mg/mL saline) was injected intravenously. Sham-operated animals underwent the same experimental procedures as described above without infusion of rose bengal and illumination. After surgery, the catheters were removed, and the animals were allowed to recover from the anesthesia and given food and water ad libitum. All animal experiments followed ethical standards, and protocols were approved by the respective government authorities.

### Exercise treatment

Forced Arm Use (FAU)-treated animals were fitted with a 1-sleeve plaster cast. The upper torso was wrapped in soft felt, and the ipsilateral forelimb was wrapped in felt and positioned in a naturally retracted position against the animal’s sternum. After a period of 4 days, the procedure was suspended for 48 hours. At day 6 of the treating period, the cast was reapplied. Animals in the voluntary exercise group (VE) were housed individually in a cage with a running wheel assembly for 10 days. Each revolution of the freely accessible wheel was electronically counted and recorded in this time period. Animals in the cage control group were housed individually in standard laboratory cages. These animals received no specific training.

### Behavioral testing

**Figure 1:**
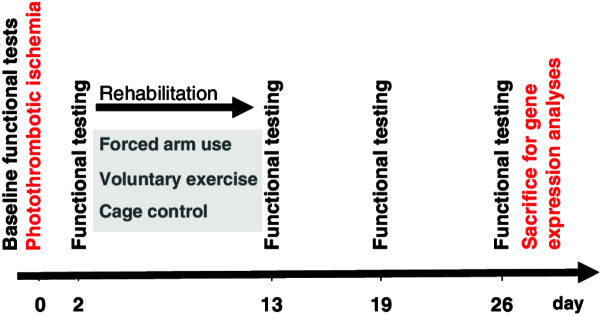
**Experimental design.** 42 Wistar rats were randomly treated with either FAU (group 1; 1-sleeve plaster cast onto unaffected limb at 8/10 days), VE (group 2; connection of a freely accessible running wheel to cage), or a cage control condition (group 3) for 10 days starting at 48 hours after photothrombotic stroke of sensorimotor cortex (day 3 to 13). Functional outcome was measured using two sensorimotor tests focused on motor control of the front paw (adhesive tape removal and cylinder tests) at baseline before ischemia (day 0), 2 days after ischemia (day 2), after the training period of 10 days (day 13), and at 3 (day 19) and 4 weeks (day 26) after ischemia by an investigator blinded to the experimental groups. Animals were sacrificed at 4 weeks after induction of stroke. For gene expression changes samples were taken from the ipsi- and contralateral cortex and the hippocampus, and hybridized to Affymetrix DNA arrays.

All animals were operated and tested in parallel (1 animal per group at once). In all animals, behavioral tests were performed at baseline before ischemia, after ischemia (24 h after operation procedure), after the training period of 10 days (day 13), and at 3 and 4 weeks after ischemia by an investigator blinded to the experimental groups. The behavioral tests after ischemia and after the training period of 10 days were performed with sufficient distance to anesthesia procedure. The experimental setup scheme is given in Figure 1.

### Adhesive removal test

The adhesive removal test was done at baseline before ischemia, after ischemia, after the training period of 10 days, and at 3 and 4 weeks after ischemia to test sensory and motor function. Initially, 2 pieces of adhesive-backed paper dots (113.1 mm^2^) were used as bilateral tactile stimuli on the dorsal paw of each forelimb. The time to remove each stimulus from the forelimbs was recorded 3 trials per day for each forepaw. Individual trials were separated by 5 minutes. Before surgery, animals were trained for 3 days.

### Cylinder test

The cylinder test was done at baseline before ischemia, after ischemia, after the training period of 10 days, and at 3 and 4 weeks after ischemia to test motor and coordinative function. The animals were not trained before ischemia. The rats were placed in a transparent Plexiglas cylinder (20 cm high, 20 cm diameter) placed on a glass table for 5 minutes and recorded on video. For analysis, the number of independent placements of the forelimbs was measured over a time period of 30 seconds. The analysis was performed off-line based on the video recording.

### Gene expression analysis

22 brains were analyzed by gene expression analysis (Cage 01–08, VE 01–07, FAU 01–07). Horizontal cryosections (8 μm) were prepared from brains of HBSS (hanks balanced salt solution)-perfused animals. Sections were thionin-stained, and laser dissection was performed with a Laser-Dissection Microscope (Leica Microsystems LMD6000, CryLaS FTSS 355–50 laser, Hitachi HV-D20 camera). Additional file 1: Figure S1 shows the relative position of the horizontal sections used for dissection, and an exemplary view on the stained hippocampus before and after laser microdissection. Per Section 3 areas were dissected: the ipsi- and contralateral cortex, and the ipsilateral hippocampus. The cortex areas for dissection were located immediately posterior to the lesion site with a diameter of 2 mm, and the corresponding area on the contralateral cortex. Per animal dissection material from 3 consecutive sections was pooled.

RNA was isolated using the Rneasy micro Kit (Qiagen, Hilden, Germany). RNA was amplified over 2 rounds using a proprietary protocol [16,17]. In brief, RNA was precipitated, resuspended, and mixed with T7-tagged dT21V oligonucleotides. 2 rounds of amplification were performed using T7-RNA polymerase. The conditions used guarantee linear amplification with low RNA input material. Biotin-marked second-round aRNA was produced with an NTP-mix composed of: Biotin-11-CTP and Biotin-16-UTP (PerkinElmer) (2 mM f.c.) and the T7–Megascript kit (Applied Biosystems/Life Technologies GmbH, Darmstadt Germany). Biotin-labelled amplified RNA (aRNA) was controlled for size distribution and amount using the Agilent 2100 Bioanalyzer with the RNA 6000 Nano LabChip kit (Agilent Technologies Deutschland GmbH, Böblingen, Germany) (Additional file 2: Figure S2). Hybridization was done with Affymetrix GeneChip® Rat Genome 230 2.0 Arrays. Arrays were analyzed by an Affymetrix GeneArray Scanner3000 (University of Mannheim, Center for medical research (ZMF)).

After array hybridization data were analyzed by Genesifter software (http://www.genesifter.net). Initial quality analysis revealed high homogeneity of hybridization signal distributions (Additional file 3: Figure S3). Data were normalized over all arrays using GC-RMA. Statistical testing was done using two-way ANOVA for the factors location and treatment. False discovery rate was controlled using the Benjamini-Hochberg procedure.

### Statistics

Experiments were performed in a completely randomized and blinded manner. Statistical analyses were done using JMP 8.01 (SAS, Cary, NC, USA). Gene expression analysis statistics were done using T-test and Benjamini-Hochberg FDR (false discovery rate). Sensorimotor measurements were analyzed with 2-way repeated-measures analysis of variance followed by the Fisher protected least significance difference test. An α error rate of 0.05 was taken as the criterion for significance.

## Results

### Forced training is superior to voluntary training for motor recovery

**Figure 2:**
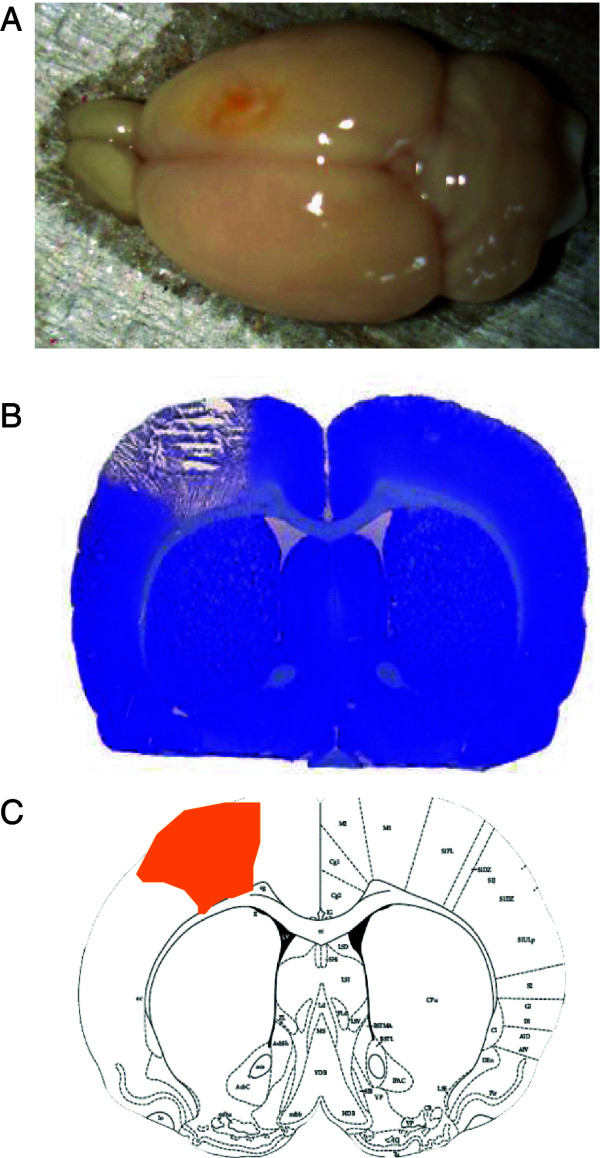
**Photothrombotic infarction of the right parietal cortex.** Shown are **(A)** a view on the brain surface with the infarcted area (see yellow colouring), **(B)** a histological stain (Thionin) of coronal sections from the rat brain in the infarct area, and **(C)** the corresponding localization to the front paw sensorimotor cortex (M1/S1) on a brain map. Note the sharp delineation of the infarct, and the intactness of the corpus callosum.

Photothrombotic ischemia resulted in all animals in a well-defined infarct in the right parietal cortex (see Figure 2). All animals displayed paresis of the left forearm. 48 rats were included in the operation procedure, five animals died. The remaining 43 animals were randomly treated with either forced arm use (FAU, induced by a cast to the unaffected arm), voluntary exercise (VE, animals allowed free access to a running wheel), or a cage control condition. In this study, infarct volumes were not determined. In previous studies we were able to show that this type of physical training had no effect on this type of small cortical infarctions [18,19].

Recovery of animals was measured up to 4 weeks following ischemia using two tests centered on motor performance in the front paws, the adhesive tape removal test, and the cylinder test. The experimental setup is shown in Figure 1.

**Figure 3:**
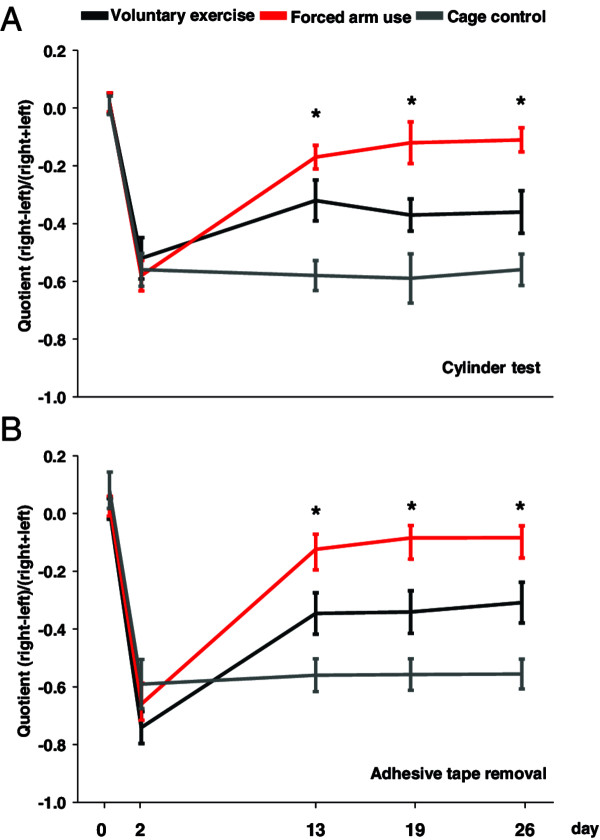
**Functional motor recovery of rats subjected to different training paradigms.** Functional improvement of the left, paretic forepaw after photothrombotic stroke measured with the cylinder test **(A)** and the Adhesive tape removal task **(B)**. Note the clear improvement of both forced (red curve) and voluntary (black curve) training compared to untreated animals (grey curve). (*p <0. 05, ANOVA, Fisher’s test).

Rehabilitative training after focal cerebral ischemia with both forced arm use (FAU) and voluntary exercise (VE) compared to cage control condition significantly improved functional recovery after focal ischemia (Figure 3). This improvement in functional neurological outcome was visible directly after the training period and remained stable over 4 weeks after ischemia. Neurological recovery was clearly better after forced training compared to voluntary running (Figure 3).

### Gene expression changes reflect the different effects of training

We analyzed three regions of bona fide interest to motor recovery processes linked to exercise: a 2 mm broad cortical area caudally adjacent to the infarct border, the corresponding area of the contralateral cortex, and the whole ipsilateral hippocampus. A total of 22 brains were analyzed, 8 from the control group, 7 from both exercise groups. Animals were randomly selected for the analysis. Gene expression was performed by laser-mediated dissection of the tissue, amplification of mRNA, and hybridization to DNA arrays.

**Figure 4:**
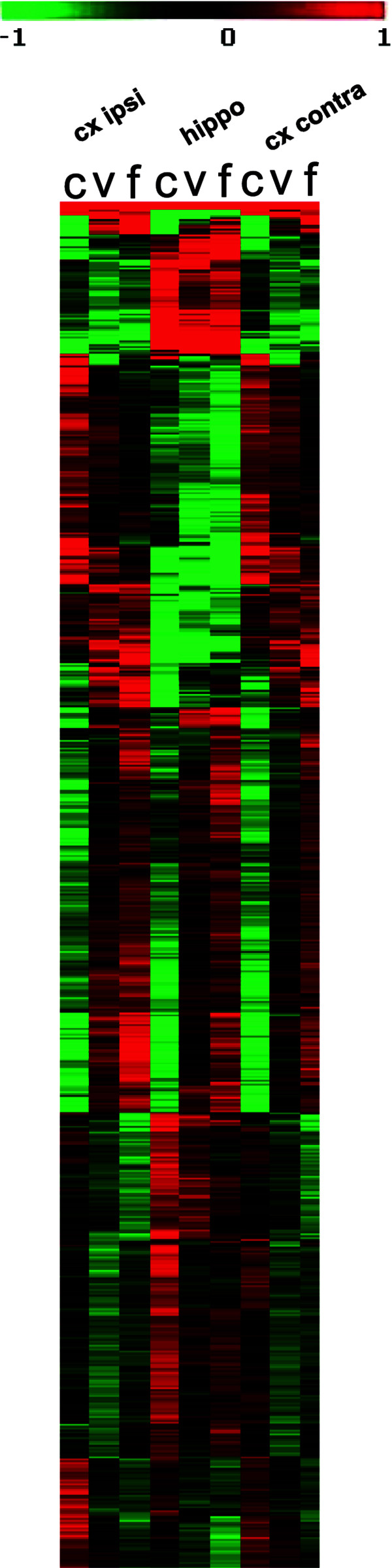
**Heat map showing an excerpt from the primary analysis of gene expression data.** Red, up regulated genes, green, down regulated genes. The regulation factors are centered over each row. Genes are clustered according to similar behavior across the experimental groups. The three exercise paradigms show clearly different patterns.

For an initial view on the data we contrasted all training groups and all locations to each other using scatter plots (Additional file 4: Figure S4). This already suggested a strong influence on gene expression of the two training praradigms in all locations examined. We then performed a 2-way ANOVA analysis with the two factors location and exercise condition. A total of 3613 probe sets were significantly influenced by the factor exercise (significance criteria: p < 0.01 after multiple testing adjustment for Benjamini-Hochberg false discovery rate) (Additional file 5: Table S1). Figure 4 shows an excerpt heat map from the initial hierarchical clustering.

**Figure 5:**
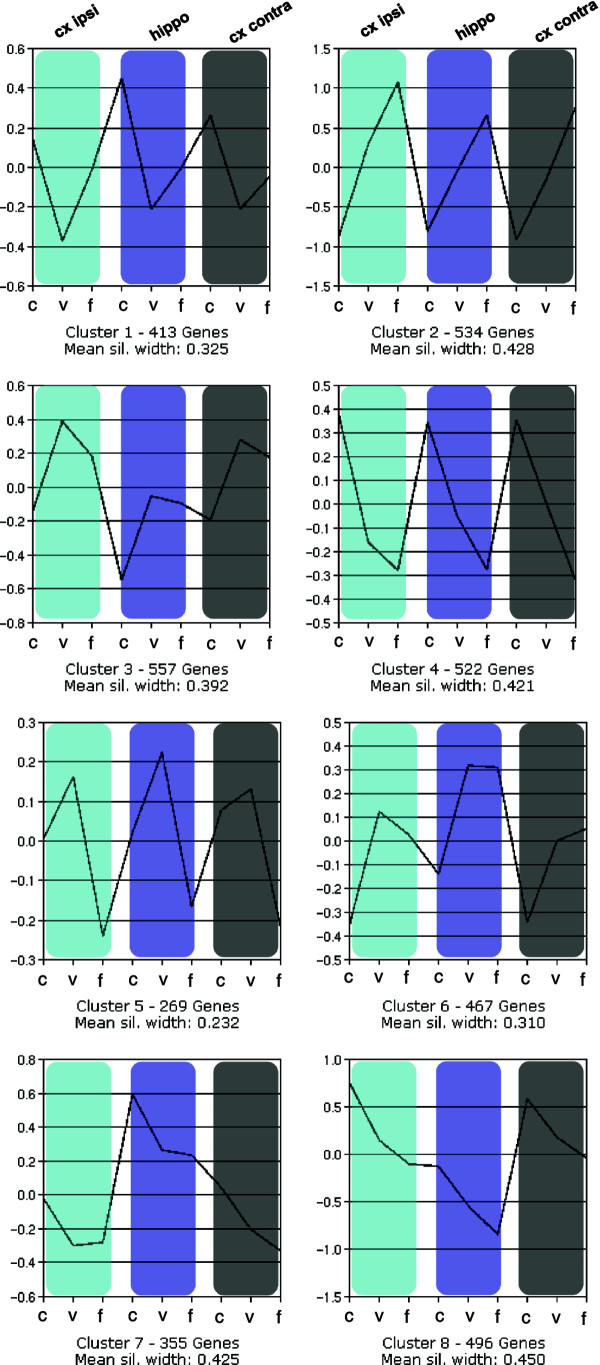
**PAM clustering of regulation patterns observed over the training paradigms and regions studied.** The gene set of 3616 differently regulated genes was subjected to the PAM algorithm clustering (patterning around medoids) (clusters = 8, average silhouette width = 0.383, distance measure used = correlation). C, control; v, voluntary and f, forced training. While most clusters follow one direction (up or down) from cage controls in the direction control – voluntary – forced training which correlates to the degree of recovery observed in the behavioral tests, a few clusters show divergent behavior for groups of genes in voluntary and forced training (cluster 3, 5, and partially 4).

To obtain more information on the patterns of regulation observed we clustered the gene set using the PAM algorithm (patterning around medoids) [20]. This revealed that there was an astonishingly high degree of similar behavior patterns of individual genes across the three brain areas sampled (Figure 5; clusters = 8, average silhouette width = 0.383, distance measure used = correlation). Indeed, there is no cluster formed where genes in the three treatment paradigms behaves fundamentally different dependent on the location the sample was taken from (i.e. in cluster 1 genes have the highest expression in the cage control animals, go down with voluntary training, and up again with forced training whereas in cluster 2 genes increase continuously in expression from control over voluntary to forced training, but have the same behavior in cortex or hippocampus). We believe that this is quite a remarkable finding to see that training influences all examined regions in the same way. Also, while many clusters indeed follow one direction (up or down) from cage controls in the direction control – voluntary – forced training paralleling the differences in behavioral recovery, a few clusters show divergent behavior for groups of genes in voluntary and forced training (cluster 3, 5, and partially 4).

**Figure 6:**
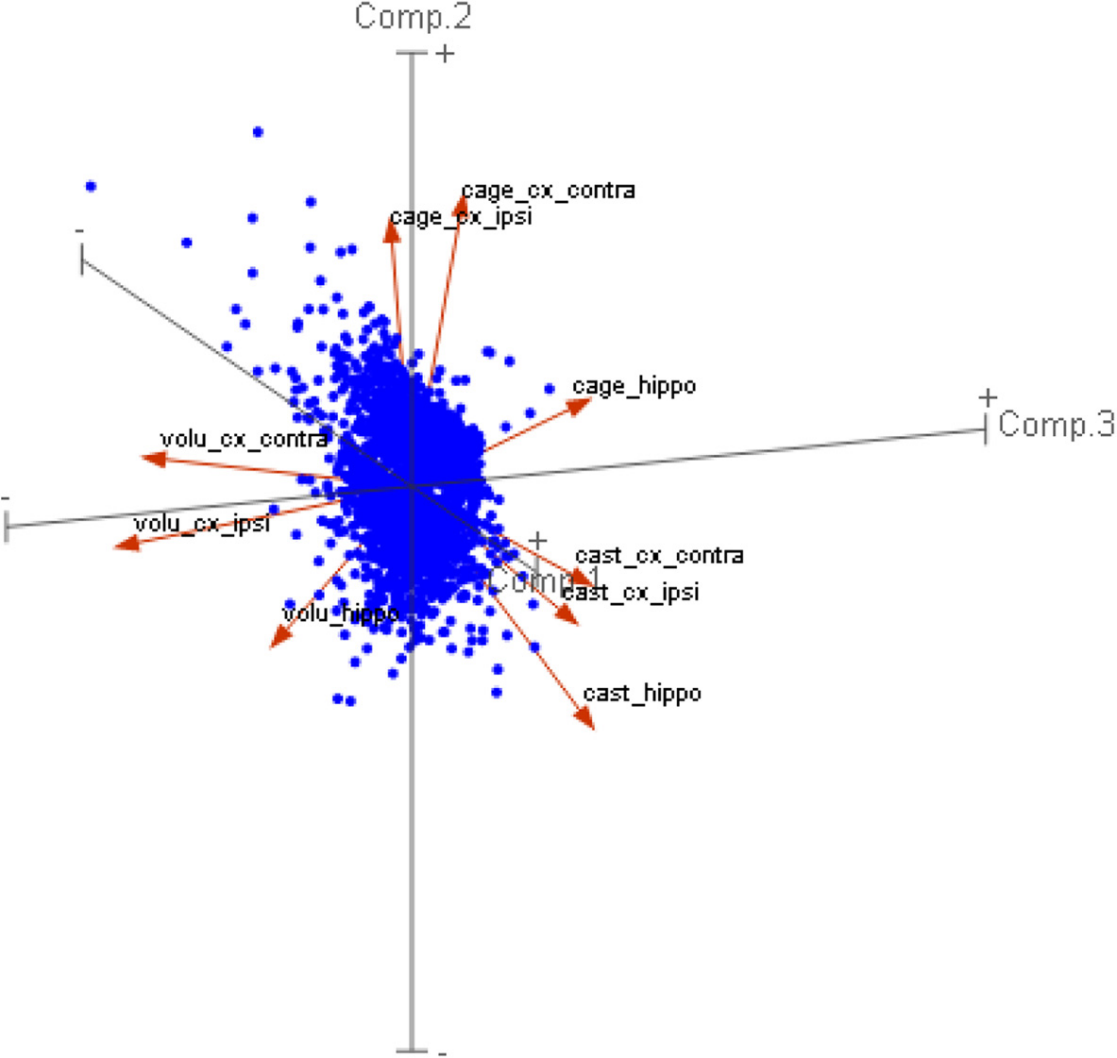
**Principal component analysis (PCA) of all genes significantly regulated.** All 3616 regulated genes were subjected to principal component analysis (PCA). Closest together are group vectors that are related by treatment, independent of location. Note that still the two cortex samples are closer together than the hippocampus vector in each treatment group.

The notion of a strong congruence of gene regulation in the three different regions examined was also underscored by a principal component analysis (PCA). Proximity of the main group vectors was determined much more by treatment than by origin of the samples analyzed (Figure 6).

To obtain information on the functional significance of the genes regulated we performed relative enrichment analyses using DAVID (http://david.abcc.ncifcrf.gov/) [21,22]. We used the functional annotation clustering tool that groups significantly overrepresented genes from different categorization systems (gene ontology, KEGG, SP-PIR etc.) in the dataset into summary categories. The following categories were identified as significant and had a relative enrichment of at least 2-fold: mitochondrion, RNA splicing/processing, cytoskeleton, ATP binding, Huntington’s/Alzheimer’s/Parkinson’s disease, zinc binding, microtubule, protein complex assembly, transcription regulation, cytoplasmic/synaptic vesicles, protein synthesis initiation, protein degradation, synapse/postsynaptic density, neuron projection, regulation of neuronal synaptic plasticity, synaptic transmission, membrane organization. The dominating themes hit by a number of clusters appear to be mitochondrion, protein degradation, transcription, and neuronal plasticity.

**Figure 7:**
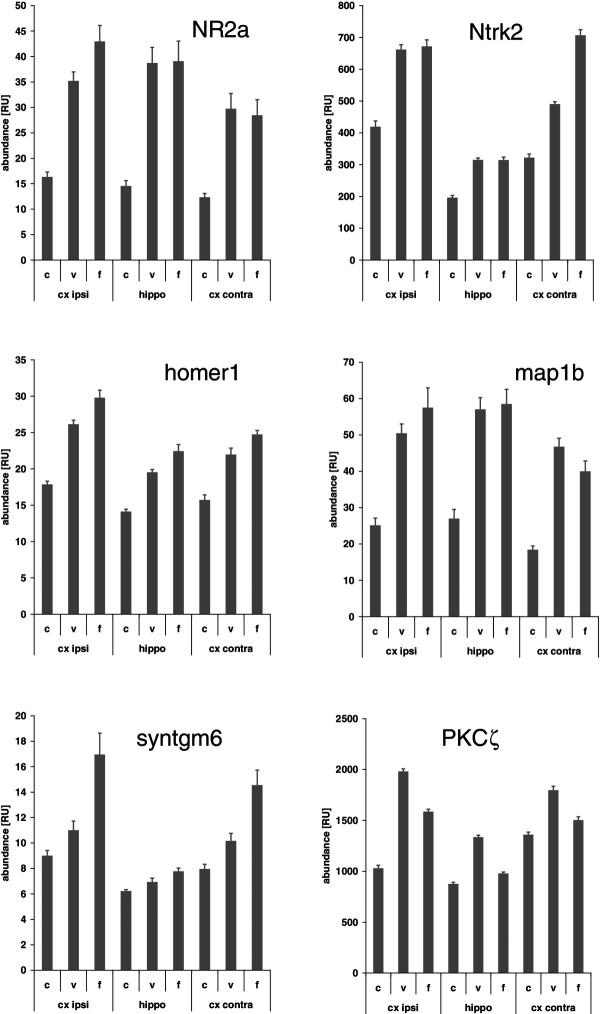
**Selection of regulation details of 6 genes linked to neuronal plasticity.** The detailed regulation data for a selection of 6 genes that have the strongest evidence-based link to learning processes and plasticity is depicted (mean +/- SEM). C, control; v, voluntary and f, forced training. Cx ipsi: cortex ipsilateral; hippo, hippocampus; cx contra, cortex contralateral.

We next concentrated on genes linked to neuronal plasticity for further analysis. We manually screened the list of genes that were at least 1.3-fold up regulated in the mean (over all three regions examined) for genes that have been experimentally shown to have significance for plasticity. The resulting list of genes together with their regulation factors is given in Table 1. This list is made up of hallmark genes for neuronal plasticity, such as the NMDA 2A receptor (NMDAR2A/GRIN2A)[23-27] (mean up regulation 2.5-fold), NTRK2/TrkB (BDNF receptor) [28-32] (mean up regulation 1.69-fold), the GRIK1/GLUR5 glutamate receptor [33-38] (mean up regulation 1.65), Grip1 [39-42] (mean up regulation 1.38-fold), homer1 [43-47] (mean up regulation 1.55-fold), or the atypical protein kinase C ζ [48-52] (mean up regulation 1.36-fold). Interestingly, we also found the mRNA for KIBRA/WWC1 up regulated (mean up regulation 1.5-fold), a protein strongly linked to human learning and memory [53-60]. The detailed regulation data for a selection of 6 genes that have the strongest evidence-based link to learning processes and plasticity is depicted in Figure 7 (mean +/- SEM).

**Table 1:** Selection of up regulated genes linked to neuronal plasticity

	**Ipsi cx**		**Hippocampus**		**Contra cx**	
**Gene title**	**Factor volu/control**	**Factor cast/control**	**Factor volu/control**	**Factor cast/control**	**Factor volu/control**	**Factor cast/control**
Receptors/postsynaptic plasticity						
Glutamate receptor, ionotropic, N-methyl D-aspartate 2A	2.16	2.63	2.66	2.69	2.41	2.31
Glutamate receptor, ionotropic, kainate 1	1.35	1.47	1.79	2.08	1.20	1.81
Glutamate receptor interacting protein 1	1.11	1.45	0.95	1.53	1.13	1.67
Neurotrophic tyrosine kinase, receptor, type 2	1.58	1.60	1.60	1.60	1.52	2.20
Homer homolog 1 (Drosophila)	1.39	2.07	1.36	1.87	1.33	2.16
Protein kinase C, zeta	1.92	1.54	1.52	1.12	1.32	1.11
Neuronal morphological plasticity						
Dynein, axonemal, light chain 1	2.40	2.96	1.91	2.52	1.78	2.39
Neurexin 1	2.09	1.88	2.30	2.44	2.30	2.50
Microtubule-associated protein 1B	2.01	2.29	2.12	2.17	2.54	2.17
Microtubule-associated protein tau	1.82	1.84	1.79	1.79	1.87	1.89
Tenascin R	1.33	2.04	1.62	2.52	1.98	
MAP/microtubule affinity-regulating kinase 3	1.76	1.61	1.32	1.23	1.44	2.45
Dynein cytoplasmic 1 light intermediate chain 1	1.27	1.14	1.78	1.53	1.34	1.29
Connectivity, pathfinding, plasticty						
Slit homolog 2 (Drosophila)	1.95	2.13	1.75	1.87	2.02	2.05
Eph receptor B2	1.39	1.40	1.26	1.58	1.39	1.44
Eph receptor B3	0.96	1.33	1.48	2.10	1.09	1.62
EphA5	1.83	2.50	1.77	2.22	1.74	2.35
Ion channel						
Sodium channel, voltage-gated, type III, beta	1.70	1.62	2.19	2.05	1.84	2.60
Presynapse						
Synaptotagmin V	1.22	1.89	1.12	1.25	1.28	1.83
Syntaxin 8	1.47	1.20	1.60	1.33	1.38	1.33

Given are the gene names and mean regulation factors in the contrast “volu/control” (voluntary training) and “cast/control” (forced arm use).

## Discussion

Physical training after photothrombotic stroke significantly and permanently improved functional recovery after stroke. Forced arm use was clearly superior to voluntary training in terms of sensorimotor recovery. Both the general effect of any physical training, as well as the difference between FAU and VE are reflected in gene expression profiles obtained from 3 different brain regions involved in recovery processes of the post-stroke brain. Gene expression changes differing between both training paradigms identify plasticity-relevant groups.

Recent studies clearly provided proof for forced arm use or constraint induced movement therapy to be effective training paradigms for improving motor function of the affected upper extremity after stroke [4-6]. A characteristic of this specific motor therapy compared to standard physical therapy is the intensiveness of the training and its high degree in standardization. Both together forces the patient to an increased active use of the paralyzed arm hereby improving gross and fine motor function. Interestingly, less intense training paradigms such as motor skill training are also suited to improve motor function of the affected extremity after a stroke compared to paradigms of less intensity such as voluntary running [61,62]. A gradually further deescalated training paradigm, where rodents control the daily intensity and the timing of the training by themselves, voluntary training, can potentially improve motor function after an insult but had in several other studies no effect (for review see [63]). Overall these findings suggest that a physical training is the more effective the more structured and intense it is.

Forced arm use or constraint induced movement therapy can principally be applied to all patients with a medium or medium-severe paresis of the arm independent of the localization of the infarction [6]. Experimental data suggest, however, that overuse in the hyperacute phase of a developing lesion might be detrimental, impair motor function, and attenuate lesion size [7-10]. These findings are corroborated by a recent clinical study, where constraint induced movement therapy applied in the early subacute phase after stroke (starting day 10 post-stroke) was not superior to standard physical therapy [64]. Moreover, an intensified constraint induced treatment arm in this study (extended treatment interval of 3 hours compared to 2 hours in the regular arm) produced an even worse outcome at the end of the observation period (90 days post-stroke). These findings illustrate that this type of treatment is highly effective but a careful selection of the timing and dosing is warranted, something that currently has just been partly explored. A biological explanation for this observation might be that induction of additional stress onto already compromised brain tissue in the periphery of a lesion may further compromise this brain tissue and finally impair recovery or exaggerate lesion size.

Forced arm use and other forced training paradigms lead to a number of processes in the brain, so called plasticity-related structural changes. Such changes include the induction of neurogenesis in the subgranular zone [65], regulation of growth factor receptors and release of their ligands such as BDNF or IGF-I [66], activation of proteins for synaptic and dendritic plasticity such as MAP-2, synapsin, or synaptophysin [19,67], and up regulation of AMPA receptors [18].

We confirm this strong influence of post-stroke training (both voluntary and FAU) on basal neuronal plasticity mechanisms, and find up regulation of NTRK2, NMDA 2a receptor, or MAP1b. Correlated to the behavioral observations, forced arm use generally leads to a stronger regulation of genes (both up – and down regulation) than voluntary training, reflecting the clinical observation that intensity and structure is a strong driver to improve motor function.

In conclusion, we have shown that intensive and structured training paradigms such as forced arm use result in the best functional motor outcome after stroke. This is reflected by a quantitative, but not qualitative, change in the gene expression program linked to recovery through training. These findings may open new approaches to further improve post-stroke rehabilitation and to develop adjunctive pharmacological therapies after stroke.

## Competing interests

The authors declare no competing interest linked to this study.

## Authors’ contributions

AS and WRS designed the study, and drafted the manuscript. AR, JM and KD performed animal experiments, OW and GV performed statistical array analyses, NG performed array hybridizations, FK, CK, ChP performed histological work, laser microdissection, RNA preparation and amplification, CP and RL were involved in data evaluation and discussions. All authors read and approved the final manuscript.

## Supplementary Material

Additional file 1: Figure S1 Shows the relative position of the horizontal sections used for dissection (A), and an exemplary view on the stained hippocampus before and after laser microdissection (B). Click here for file

Additional file 2: Figure S2 Electropherogram of amplified RNA (Agilent Bioanalyzer). Note the excellent size distribution of the amplified RNA. Ladder size (Agilent RNA 6000): 0.2 kb, 0.5 kb, 1.9 kb, 2.9 kb, 4.0 kb, 6.0 kb. Click here for file

Additional file 3: Figure S3 Quality control for the distribution of signal intensities on the Affymetrix array. The distribution is highly homogenous over all groups with no outliers. Click here for file

Additional file 4: Figure S4 Overview of all two-way comparisons as scatter plots, red: up regulated genes, green: down regulated genes (Welch’s t-test, false discovery rate correction: Benjamini Hochberg, threshold: 1.5). “cast” = forced arm use, “volu” = voluntary exercise, “cage controls” = control animals. It is obvious that far fewer genes are changed between the two training paradigms than between each training paradigm and the control animals. Click here for file

Additional file 5: Table S1 Given are 3613 probe sets that were significantly influenced by the factor exercise (significance criteria: p < 0.01 after multiple testing adjustment for Benjamini-Hochberg false discovery rate). Numbers in the column titles signify different exercise paradigms in the 3 different locations: 1–3, cortex ipsilateral (1 = control, 2 = voluntary, 3 = forced training); 4–6, hippocampus (4 = control, 5 = voluntary, 6 = forced training); 7–9, cortex contralateral (7 = control, 8 = voluntary, 9 = forced training). “Mean” are the log2 transformed values, with “SEM” being the standard error of the mean for each group. “Mean norm” are the non-transformed intensity values for each group. “gene identifier”, “gene title” etc. give information for the identities of the probe set. Click here for file
