# Supplementary figures and images for: Forced arm use is superior to voluntary training for motor recovery and brain plasticity after cortical ischemia in rats
Source: Exp Transl Stroke Med. 2014 Feb 14;6:3. doi: 10.1186/2040-7378-6-3 (PMC3937028; doi:10.1186/2040-7378-6-3)

## Slide 1
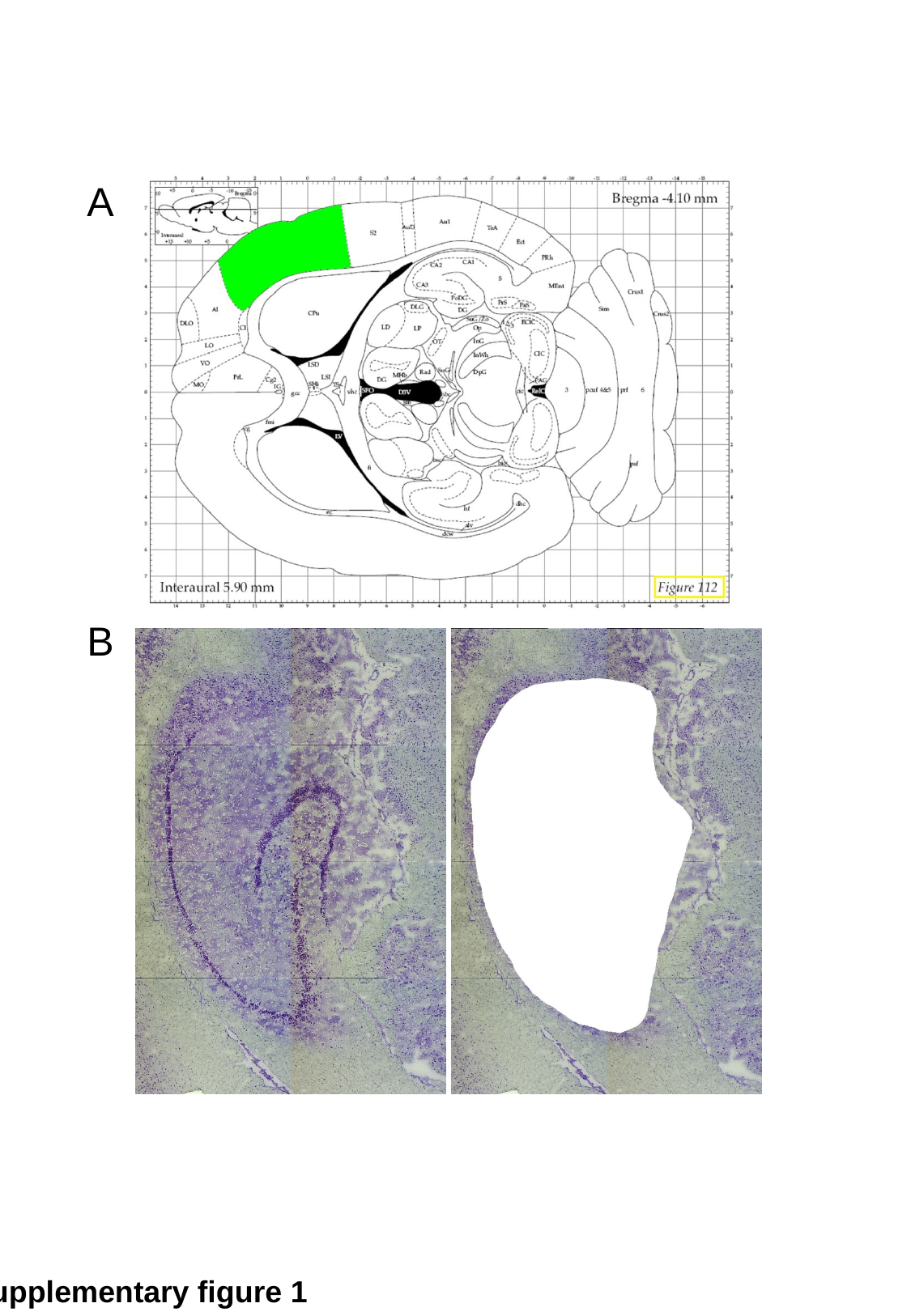

A
B

Supplement: Additional file 1: Figure S1 — Shows the relative position of the horizontal sections used for dissection (A), and an exemplary view on the stained hippocampus before and after laser microdissection (B). [file 2040-7378-6-3-S1.pptx]

## Slide 1
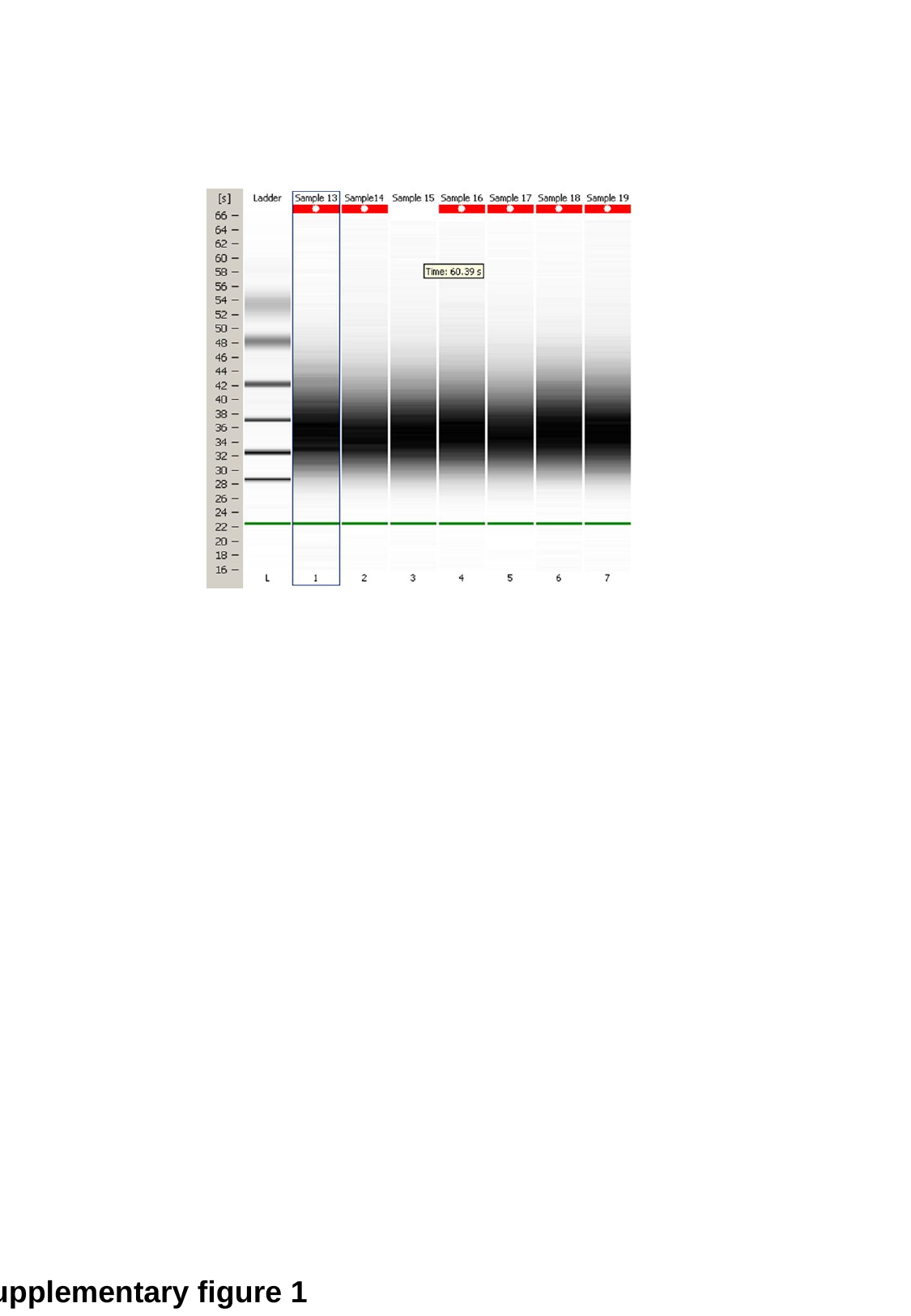

Supplement: Additional file 2: Figure S2 — Electropherogram of amplified RNA (Agilent Bioanalyzer). Note the excellent size distribution of the amplified RNA. Ladder size (Agilent RNA 6000): 0.2 kb, 0.5 kb, 1.9 kb, 2.9 kb, 4.0 kb, 6.0 kb. [file 2040-7378-6-3-S2.pptx]

## Slide 1
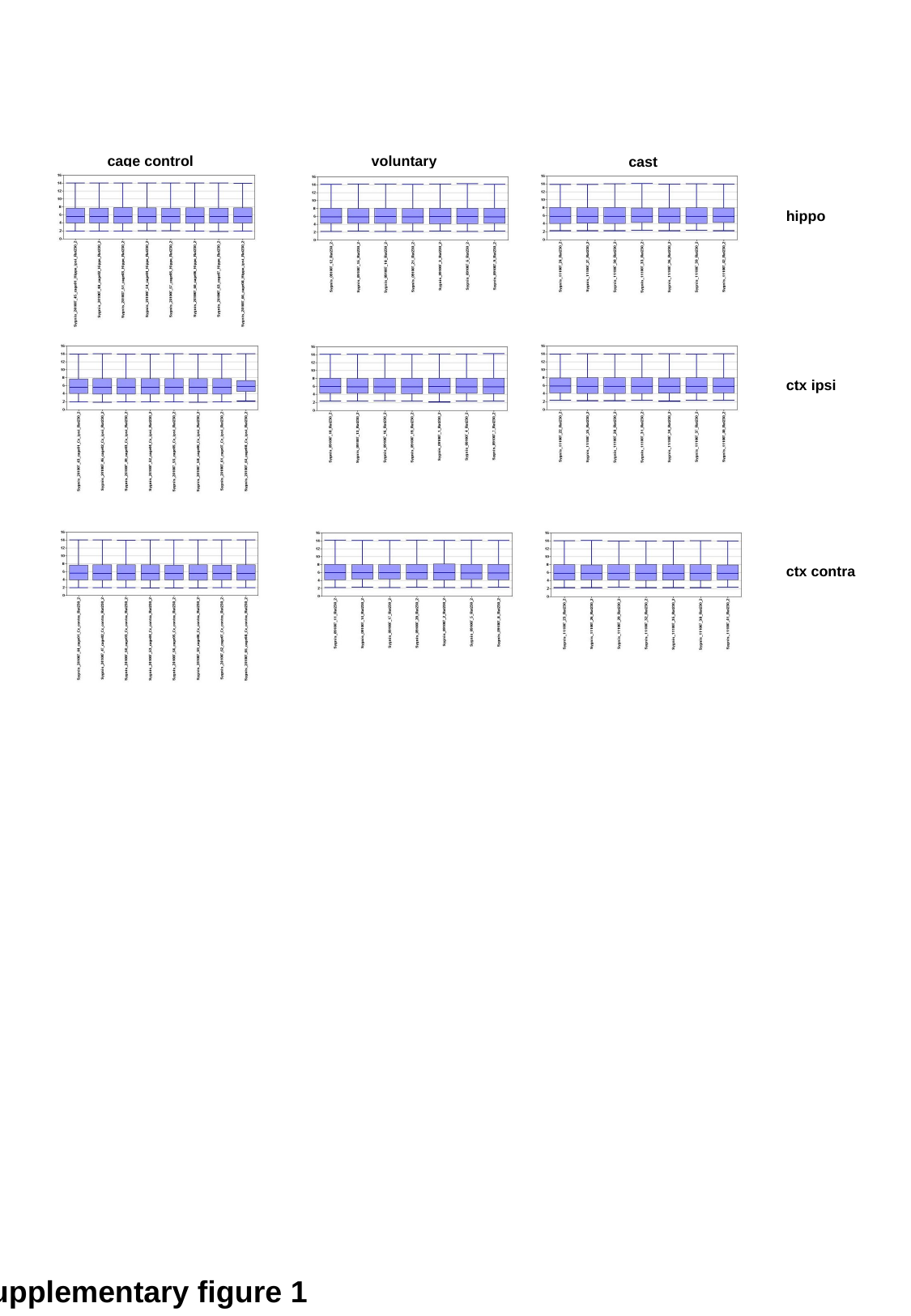

Supplement: Additional file 3: Figure S3 — Quality control for the distribution of signal intensities on the Affymetrix array. The distribution is highly homogenous over all groups with no outliers. [file 2040-7378-6-3-S3.pptx]

## Slide 1
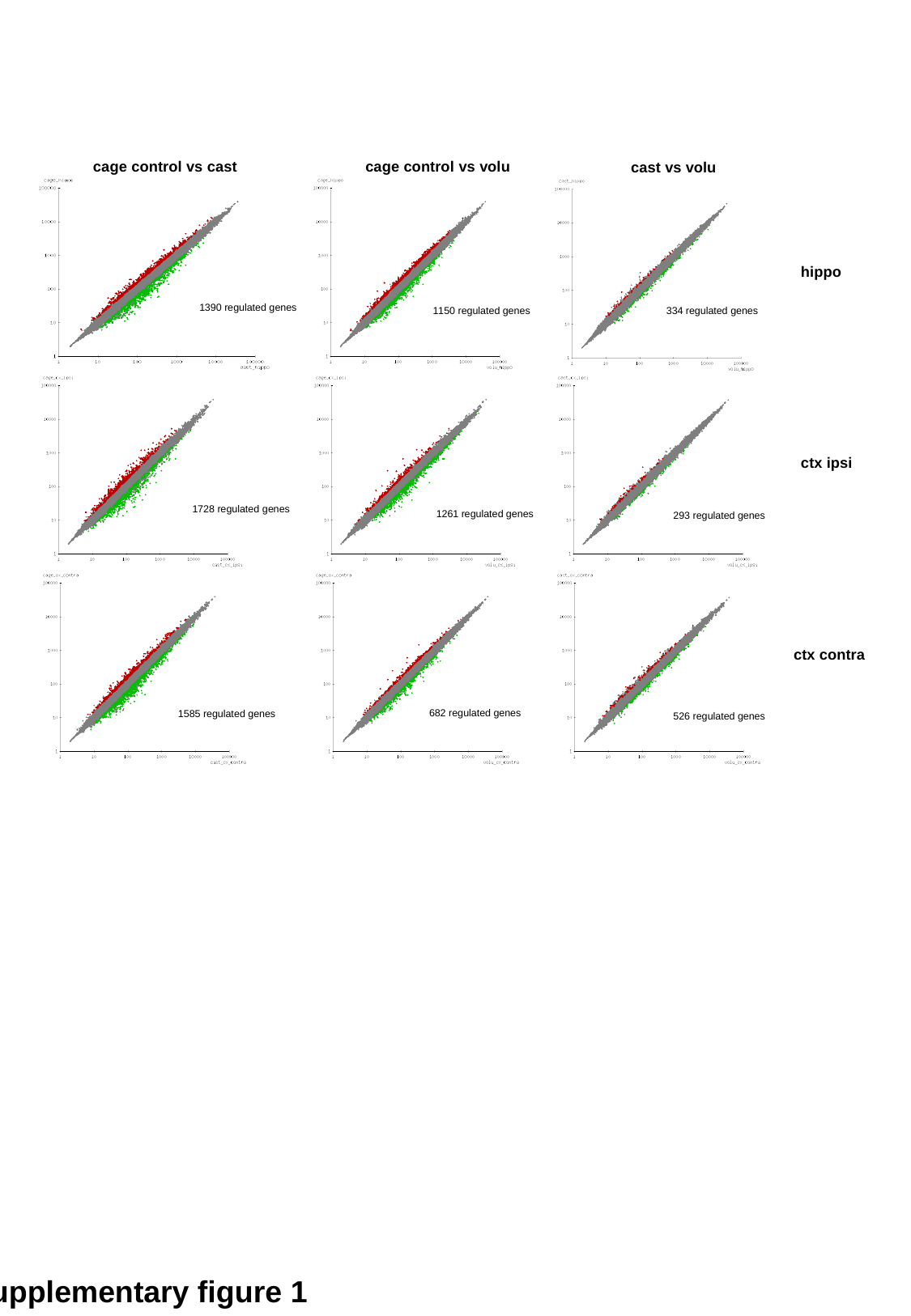

Supplement: Additional file 4: Figure S4 — Overview of all two-way comparisons as scatter plots, red: up regulated genes, green: down regulated genes (Welch’s t-test, false discovery rate correction: Benjamini Hochberg, threshold: 1.5). “cast” = forced arm use, “volu” = voluntary exercise, “cage controls” = control animals. It is obvious that far fewer genes are changed between the two training paradigms than between each training paradigm and the control animals. [file 2040-7378-6-3-S4.pptx]
